# The role of rehabilitation care workers in South African healthcare: A Q-methodological study

**DOI:** 10.4102/ajod.v8i0.537

**Published:** 2019-10-29

**Authors:** Shamila Gamiet, Michael Rowe

**Affiliations:** 1 Department of Physiotherapy, University of the Western Cape, Cape Town, South Africa

**Keywords:** community, community-based rehabilitation, community health workers, intermediate care, participation, primary healthcare, South Africa

## Abstract

**Background:**

The South African Department of Health identified the need to train a new cadre of community health worker (CHW) in the field of rehabilitation as part of their 2030 Health Plan that aims to improve primary healthcare (PHC) and community-based rehabilitation (CBR). Community health workers can be effectively utilised in CBR if their role is understood and their potential is not limited by professional protectionism and scepticism. A clear understanding of the scope of practice of a new cadre will minimise resistance by health professionals.

**Objectives:**

The aim of this study was to explore rehabilitation health professionals’ perception of the role of the new cadre, called rehabilitation care workers (RCWs), in South African healthcare.

**Methods:**

Q-methodology was used to gather and interpret the data. A convenient sample of 16 health professionals participated in the study. Participants ranked statements about the role of the RCWs from strongly agree to strongly disagree. Data were entered into PQMethod software program for statistical and factor analysis.

**Results:**

Two factors emerged. Participants loading onto Factors 1 and 2 were of the opinion that RCWs’ role would be to strengthen PHC and CBR and to promote participation of people with disabilities (PWD) in intermediate care and community.

**Conclusion:**

Rehabilitation health professionals’ positive perception of the new cadre is encouraging so that it could ensure their effective utilisation in CBR. Rehabilitation care workers were perceived as capable of enhancing the lives of PWD by ensuring inclusive development.

## Introduction

People with disabilities (PWD) often come from vulnerable communities and experience difficulties with everyday functioning. They struggle to access health and rehabilitation, education and employment opportunities and this leads to poorer health outcomes, lower education achievements and higher rate of unemployment in comparison to people without disabilities (World Health Organization & World Bank 2011). One of the main barriers that PWD face is poor access to healthcare services (World Health Organization & World Bank 2011). It is estimated that only a small percentage of PWD have access to rehabilitation and basic health services (World Health Organization & World Bank 2011). The World Health Organization (WHO) has identified community-based rehabilitation (CBR) as a comprehensive framework for addressing the needs of PWD in compliance with the principles of primary healthcare (PHC) (WHO 2010). Community-based rehabilitation thus improves PWD access to rehabilitation services.

However, due to a shortage of skilled rehabilitation health professionals, effective implementation of CBR programmes requires additional health workers (Gupta, Castillo-Laborde & Landry 2011). The WHO recommended that community health workers (CHWs) be utilised in CBR to improve access to rehabilitation and health services. Community health workers have been defined by the WHO as members of the communities in which they work, selected by the communities, supported by the health system and who have shorter training periods than qualified health professional workers (WHO 1989). Community health workers have been recognised globally as playing a vital role in improving access to health services in order to strengthen PHC and CBR (Friedman 2002; Lorenzo, Motau & Chappell 2012). Their role is to deliver rehabilitation services because it is expensive and difficult to get health professionals to work in the community (Rule, Lorenzo & Wolmarans 2006). In South Africa, PWD in rural areas have benefitted from CBR programmes utilising CHWs. These benefits include physical rehabilitation, education on rehabilitation, emotional support, counselling, access to resources and assistive devices and, most importantly, reintegration into the community (Dawad & Jobson 2011). Community health workers made a significant impact in the lives of PWD through home visits, exercise, assistive devices and training in activities of daily living, resulting in an increase in independence, better social integration and mobility. Community health workers also had a positive impact on communities by changing the negative attitude towards PWD (Chappell & Johannsmeier 2009).

According to Dovlo (2004) CHWs can be effectively utilised in CBR if their role is understood and their potential is not limited by professional protectionism and scepticism. A clear understanding of the scope of practice of a new CHW will minimise resistance by health professionals (Hugo 2005; Rule et al. 2006). In South Africa the role of CHWs may be limited due to a lack of understanding of their capabilities. This could be due to poor input on CBR and the role of CBR personnel during their professional training (Bury 2005; Lehmann & Gilson 2012). Health professionals should also provide supportive supervision, guide and monitor CHWs and facilitate teamwork to ensure quality care (Chappell & Johannsmeier 2009). They must ensure that CHWs execute tasks at acceptable standards to ensure better health outcomes (Freeman et al. 2012). This enhances the credibility of CHWs by clarifying their roles and by ensuring they can address the problems of PWD (Freeman et al. 2012; Jaskiewicz & Tulenko 2012). A lack of knowledge of CBR and its cadres can therefore lead to poor supervision and limitation of the CHWs’ role in CBR (Chappell & Johannsmeier 2009; Lehmann & Gilson 2012). Sufficient support for any new cadre of CHW is crucial in developing a patient-centred approach, integrated provision of care, continuity of care and a holistic approach to treatment which is on-going (Crigler, Gergen & Perry 2013; Jaskiewicz & Tulenko 2012). It is therefore important that rehabilitation health professionals accept new cadres of worker as this is essential in the successful implementation of CBR programmes.

The South Africa’s National Department of Health (DOH) is committed to addressing the needs of PWD by strengthening PHC services and community-based services (CBS) (Western Cape Government Health 2014). Primary health ensures that PWD live a socially and economically productive life, allowing for employment, education, and engagement in family and community activities. The South African DOH therefore identified the need to train a new cadre of CHW in the field of rehabilitation as part of their 2030 Health Plan that aims to improve PHC and CBR. This new cadre with a new skill set will be able to work across the health, education, livelihoods, social and development sectors thus ensuring effective implementation of CBR in South Africa (Mannan et al. 2012). It was therefore recommended that the new cadre of rehabilitation care workers (RCWs) should have mixed skills so as to address the functional abilities of an individual. These skills included self-care, playing, working, learning, communicating, hearing and mobility (MacLachlan, Mannan & McAuliffe 2011; Rule 2013).

In 2012, a pilot project was commissioned and funded by the South African DOH in the Western Cape (DOHWC) to train 30 RCWs. The vision of this pilot training programme was to upgrade the skills of current CHWs to become recognised members of the PHC team. The new cadre was CHW renamed to RCW. The CBR guidelines, recommended by the WHO in 2010, provided the conceptual framework for the training curriculum. The RCWs were given selected knowledge and skills on physiotherapy, occupational therapy and speech therapy to equip them to support and care for PWD in two underserved districts in the Western Cape. However, this was not the first time South Africa trained community rehabilitation workers (CRWs). In the late 1980s, representatives of speech and hearing therapy, occupational therapy and physiotherapy discussed the need to implement CBR programmes and to train CRWs. As a result, in the 1990s three training programmes were set up for CRWs: (1) run by South African Christian Leadership Assembly (SACLA) Health in Khayelitsha, Cape Town, (2) run by the University of Witwatersrand and Tintswalo Hospital in Acornhoek and (3) by Alexandra Health Centre in Johannesburg (Rule et al. 2006).

Unfortunately, the training of CRWs was abandoned due to increasing reluctance by the South African DOH to support personnel with multidisciplinary skills (Concha 2014). Although this current pilot runs the same risk, the success of this pilot project is important as it will establish the basis for future training of RCWs in South Africa. Rehabilitation health professionals provide the main link between RCWs and the health system. In order to provide quality healthcare and to ensure the success of CBR, rehabilitation health professionals need to understand the role of RCWs so as to support, motivate and mentor them (Chappell & Johannsmeier 2009; Jaskiewicz & Tulenko 2012). Understanding the opinions of rehabilitation health professionals, as key stakeholders in the health system, will identify how well RCWs will be utilised in CBR. Therefore, the aim of this study was to explore rehabilitation health professionals’ perceptions of the scope of practice of RCWs in South African healthcare.

## Methodology

### Design

Q-methodology was used to gather and interpret the data. Q-methodology is a mixed method approach to research as it involves elements of quantitative and qualitative analysis in systematically studying subjectivity (Ramlo 2016). This methodology was invented by British physicist–psychologist William Stephenson in 1953, who was interested in finding a way to explore the subjectivity of an issue (Herrington & Coogan 2011; Van Exel & De Graaf 2005). Studies that use Q-methodology are helpful in exploring opinions and preferences that can have an impact on behaviour (Brown 1993). As this study explored rehabilitation health professionals’ perception of the role of RCWs in the South African healthcare, Q-methodology was identified as a suitable research method to analyse the viewpoints of the participants.

### Study population and sampling

The total study population included 27 rehabilitation health professionals who engaged directly with the RCWs in the clinical settings during their work-integrated practice learning module. They were full-time and part-time physiotherapists, occupational therapists, speech therapists, physiotherapy assistants and occupational therapy technicians employed at intermediate care facilities and all the clinical educators who supervised the RCWs in the clinical setting. Intermediate care facilities refer to inpatient institutions that provide healthcare to patients who are not critically ill but still need support to carry out activities of daily life after an episode of illness. Although the total population of 27 rehabilitation health professionals were invited to participate in this study, only 16 participants consented to take part.

### Procedure

Q-methodology has two components. The first component is the collection of data to inform the Q-concourse. The concourse refers to the flow of communicability surrounding any topic in the ordinary conversation, commentary and discourse of everyday life (Brown 1993). A Q-concourse consists of a selection of statements regarding the topic. In this study, the data required to develop the concourse were collected from focus group discussions, document analysis and a review of the relevant literature. Several statements of opinion emerged. These statements are referred to as the Q-set.

Data collection for the second component of a Q-study is called Q-sorting. Participants were provided with written and verbal instructions on the Q-sorting process. Q-sorting was conducted collectively at one adult intermediate care facility and one paediatric intermediate care facility in the Western Cape, where RCWs were placed for their clinical training. Some participants completed the Q-sorting individually in their own time, and then electronically returned their completed Q-data score grids to the researcher. The participants ranked the Q-set on a data scoresheet in the form of a grid. The data scoresheet is a diagram consisting of columns in which the statements, obtained from the Q-concourse, were ranked. The participants were instructed to read all the statements carefully and then sort the statements into three categories, namely statements they agreed with, statements they disagreed with and statements they felt neutral about. The participants took the statements which they agreed with, and then ranked each statement from ‘strongly agree’ to ‘agree somewhat’ on the data scoresheet. Statements were ranked from +1 to +4. Statements that participants strongly agreed with were ranked +4 on the data scoresheet. The participants then took the statements which they disagreed with and ranked these statement from ‘strongly disagree’ to ‘disagree somewhat’ on the data scoresheet. Statements were ranked from −1 to −4. Statements that participants strongly disagreed with were ranked −4 on the data scoresheet. The statements which they did not have an opinion on were placed in the neutral column with numerical value of 0. Participants explained in writing on their data scoresheets why they strongly agreed and strongly disagreed with the statements they ranked at the extreme ends (that is +4 and −4). After the Q-sorting was completed, the participants reviewed how they had ranked the Q-set and could make changes if they so wished. This ensured that the participants’ personal viewpoints were accurately portrayed. A completed data scoresheet is called a Q-sort and represents the raw data.

### Data analysis

PQMethod software, which is a statistical programme tailored to the requirements of Q-studies, was downloaded from the internet for the statistical and factor analysis of the Q-data. The programme aggregated the data into factored sets. In the data analysis process, the correlation matrix of all Q-sorts (the completed data scoresheets) was calculated. This showed the level of agreement or disagreement between each of the participants in this study. The statistical method of factor analysis was used to identify common points of view among Q-sorts. In Q-factor analysis, the correlations between persons as opposed to variables are factored. It determined which sets of people clustered together. The statements and the 16 individual Q-sorts were entered into the PQMethod programme. A Centroid analysis was selected to extract factors. The resulting final set of 16 Q-sorts loaded onto two factors. These loadings represented the extent to which each Q-sort was associated with each factor.

A study limitation identified was the potential for bias as one of the researchers had some involvement with the RCW training programme.

### Ethical considerations

Permission to conduct this study was obtained from the Humanities and Social Sciences Research Ethics Committee of the University of the Western Cape (registration number: 13/10/38). Permission was obtained from all participants before commencing the research. The personal information and the names of the participants were not disclosed in the reporting of the findings and pseudonyms were used thereby ensuring anonymity. All data gathered were treated confidentially.

## Results

The two factors that emerged from this study were named according to the participants’ viewpoints of the role of RCWs in South African healthcare that were strongly featured. Factor 1 was named ‘Strengthen CBR’ and Factor 2 was named ‘Promoters of participation’. These two factors were significantly different with *p* < 0.01. Nine participants loaded onto Factor 1 and 7 participants loaded onto Factor 2 which is outlined in Table 1.

**TABLE 1:** Number of factors identified and the number of participants loading onto each factor.

Variable	Factor 1: Strengthen CBR	Factor 2: Promoters of participation
Number of defining variables (number of participants)	9	7
Average reliability coefficient	0.800	0.800
Composite reliability	0.973	0.966

CBR, community-based rehabilitation.

### Factor 1: Strengthen community-based rehabilitation

Participants in Factor 1 agreed with the statements outlined in Table 2 and ranked these statements at +4. The positive sign indicates the agreement and the numerical value indicates the strength of the agreement. Statements ranked at +4 are statements that the study participants strongly agreed with.

**TABLE 2:** The statements that participants loading onto Factor 1: ‘Strengthen CBR’ strongly agreed with.

Statement	Rank score
RCWs should work in both intermediate care and community under the supervision of a qualified health profession	+4
RCWs have a role in promoting participation of clients in the community	+4
RCWs will strengthen rehabilitation services across the health platform	+4

CBR, community-based rehabilitation; RCW, rehabilitation care worker.

Nine of the 16 participants loaded onto Factor 1. These nine participants were of the opinion that RCWs will strengthen rehabilitation services in intermediate care and in the community and will assist in promoting the participation of clients in the community, and they must be supervised by qualified health professionals.

The participants in Factor 1 strongly disagreed with the statements outlined in Table 3 ranking the statements at −4. The negative sign indicates the level of disagreement and the numerical value indicates the strength of disagreement.

**TABLE 3:** The statements that participants loading onto Factor 1: ‘Strengthen CBR’ strongly disagreed with.

Statement	Rank score
RCWs were clear of their role in the workplace and were therefore assertive when executing tasks delegated to them.	−4
As a health professional, I will not benefit from having an RCW working at my health facility.	−4

CBR, community-based rehabilitation; RCW, rehabilitation care worker.

The nine participants who loaded onto Factor 1 felt that the RCWs were not sure of their role in intermediate care and as a result the RCWs lacked confidence in performing tasks delegated by the health professionals. Participants felt that it would be beneficial to have an RCW employed at their health facility as they disagreed with the statement that they will not benefit from having the RCWs working there.

### Factor 2: Promoters of participation

Participants in Factor 2 agreed with the statements outlined in Table 4 and ranked these statements at +4 where the positive sign indicates the agreement and the numerical value indicates the strength of the agreement.

**TABLE 4:** The statements that participants loading onto Factor 2: ‘Promoters of participation’ strongly agreed with.

Statement	Rank score
RCWs should work in both intermediate care and community setting under the supervision of a qualified health professional	+4
RCWs have a role in promoting participation of clients in the community	+4
RCWs worked better in structured environments	+3
RCWs will strengthen rehabilitation services across the healthcare platform	+3

RCW, rehabilitation care worker.

Seven of the 16 participants loaded onto Factor 2 and strongly agreed, as did the participants loading on Factor 1, that RCWs should be included in the healthcare system at both intermediate care level because they worked well in structured settings and in the community where they would promote the participation of patients in their activities of daily living. Participants perceived intermediate care facilities as structured environments. This in turn would allow RCWs to assist in strengthening rehabilitation services across the health platform.

Participants in Factor 2 disagreed with one statement only outlined in Table 5 and ranked this statement at −4.

**TABLE 5:** The statement that participants loading onto Factor 2: ‘Promoters of participation’ strongly disagreed with.

Statement	Rank score
As a health professional, I will not benefit from having an RCW working at my health facility	−4

RCW, rehabilitation care worker.

The seven participants loading onto Factor 2 shared the same opinion as those participants loading onto Factor 1 that health professionals would benefit from having an RCW employed at their health facility. This is deduced from the statement above which participants disagreed with.

Participants were of the opinion that there is a definite place for RCWs in intermediate care settings and that it would be beneficial to have RCWs employed at intermediate care centres. Participants elaborated on why they felt they would benefit from having RCWs at their health facility on their Q data score grids. The following are examples of the participants’ responses:

‘I will definitely benefit from having an RCW at my facility. Nursing staff are not always able to follow through on activities in the ward whereas the RCW is able to do so. Positioning in seating devices and positioning of splints are not always managed well by nursing staff thus the RCW is able to correct a child’s position in the buggy and make sure splints are worn correctly.’ (P1, female, 37 years old, occupational therapist)

‘I have already experienced the advantage of giving specific tasks and roles to the RCW working at my facility and have seen how this changed and benefitted in the patient’s overall care and continuation of care, especially tapping into their cultural, community knowledge and to help with language barriers (e.g. Xhosa speaking clients).’ (P2, male, 33 years old, physiotherapist)

‘I feel there is a place for RCWs in our health system as they spend more quality time engaging with clients, families, understand contextual factors better and are constantly visible in communities. RCWs proved to fit well into intermediate care centres. They were able to adapt to their environment and relate better to the clients as they come from communities. They interacted and engaged with families and this is similar to what they did in community.’ (P3, female, 52 years old, occupational therapist)

The results of this Q study showed that health professionals in the Western Cape perceived that RCWs’ role would be able to strengthen CBR and promote participation of PWD in the community and in intermediate care.

## Discussion

In South Africa, CBS have two service elements, namely home and community-based care, and intermediate care. These two elements are vital in strengthening the continuity of care and person-centred care towards achieving South Africa’s 2030 healthcare vision. In line with this vision, RCWs were introduced into the health system as part of an interdisciplinary rehabilitation team.

The major theme that emerged from this Q-study was the perceived role of RCWs in the South African healthcare. Rehabilitation health professionals expressed their strong support for the utilisation of RCWs in intermediate care and in the community. RCWs would be assisting with the continuum of care of patients in the Western Cape by extending health services in underserved communities thereby improving the quality of life of PWD (Rule 2013). People with disabilities are often excluded from health, education, employment and social services which in turn can worsen disability and poverty (World Health Organization & World Bank 2011). However, through CBR programmes, the RCWs in the Western Cape would be able to focus on rehabilitation to address the difficulties faced by PWD who often struggle to access health services (Lorenzo et al. 2012). The RCWs would be able to assist PWD by breaking down barriers which would otherwise hinder their ability to enjoy social integration. This is supported by Friedman (2002) and Lorenzo et al. (2012) who reported that CRWs have a vital role in improving access to health services. Binken, Miller and Concha (2009) also found that CRWs provided valuable services to patients with a range of impairments, and performed tasks such as accessing resources, referrals, screening and assessment, individual and group treatment, and provision of appropriate assistive devices and techniques to facilitate interaction in communities. Similarly, in a study performed in Botswana, Malawi and South Africa, it was found that community disability workers (CDWs) achieved social inclusion for PWD across the lifespan. These CDWs worked towards improving the health and educational opportunities of PWD, strengthening their ability to obtain a livelihood and empowering them and their families to understand their human rights in society (Van Pletzen, Booyens & Lorenzo 2014). CRWs were also described by Lorenzo et al. (2015) as critical change agents in improving disabled youths’ access to health and education resources as CRWs were aware of the needs of disabled youth and worked towards integrating them into existing services. Despite significant contributions that CRWs make, they struggle to gain recognition from health professionals and social development practitioners as few higher education institutions consider career pathways for CRWs (Lorenzo et al. 2015).

Rehabilitation health professionals in this study reported that there was a definite role for RCWs in the community. They perceived RCWs as being capable of assisting PWD to become active participants within their community by ensuring reintegration. RCWs can deliver rehabilitation services in communities because it is expensive and difficult to get health professionals to work in the community (Rule et al. 2006). RCWs were also more comfortable working in the households of PWD as they had prior work experience in this setting. This provided further support by the health professionals for RCWs to work in the community as they would be able to continue with treatment and rehabilitation after discharge from hospital. The RCWs would also be able or follow up on patients seen at community health centres thus contributing to patient-centred approach to healthcare (Hugo 2005; Rule et al. 2006). Furthermore, Chappell and Johannsmeier (2009) reported that community rehabilitation facilitators (CRFs) made a significant impact in the lives of PWD through home visits, exercise, assistive devices and training in activities of daily living resulting in an increase in independence, better social integration and mobility.

Working in intermediate care was a new experience for the RCWs as they had only worked in community settings before the pilot project. Rehabilitation health professionals in this study agreed that RCWs worked well in the structured environments of intermediate care. They felt that the RCWs are capable of following work schedules and programmes which are drawn up for them. Rehabilitation health professionals felt that RCWs themselves were not sure of their role in intermediate care and therefore they were not assertive when executing the tasks delegated to them. This could imply poor health outcomes if patients are not effectively managed. However, this study found that the rehabilitation health professionals had a positive perception of RCWs implying support for RCWs in intermediate care. Rehabilitation health professionals indicated their support for RCWs working under their direct supervision, performing tasks which have been delegated by them. It is important that RCWs are well supervised and guided by health professionals so that they can adequately address the needs of PWD and communities thus ensuring good health outcomes (Crigler, Gergen & Perry 2013; Jaskiewicz & Tulenko 2012). It could be expected that rehabilitation health professionals will provide efficient support for RCWs thus facilitating their successful integration and utilisation in CBR. Chappell and Johannsmeier (2009) reported that rehabilitation health professionals need to accept a new cadre as it is essential in the successful implementation of CBR programmes.

## Conclusion

This study concludes that rehabilitation health professionals in the Western Cape perceived RCWs as capable of strengthening PHC and CBR across the service platform by extending health services to PWD both in intermediate care and in the community. Rehabilitation health professionals felt that RCWs can ensure the inclusive development of PWD in society. These positive perceptions are encouraging and could imply that RCWs will receive efficient support and supervision from rehabilitation health professionals thereby ensuring their effective utilisation in CBR programmes. However, in order to ensure the sustainability of CBR in South Africa, it is imperative that CBR programmes, RCWs and rehabilitation health professionals are well supported by the national government. The current training project of a new cadre with a new skill set, capable of addressing the needs of PWD across their lifespan, can be seen by the South African National DOH as successful. The DOH should therefore commit to training and supporting RCWs so as to extend rehabilitation services to more marginalised communities. It is further recommended that the South African DOH evaluate and monitor RCWs and work towards upgrading their knowledge and skills through continuous education workshops. Rehabilitation health professionals can also be supported through continuing professional development workshops aimed at understanding all aspects of CBR and its cadres. This would take South Africa one step closer to achieving its 2030 health vision which aims to ensure access to health and rehabilitation for all.
